# Splenic Vein Obstruction Due to a Solitary Echinococcal Splenic Cyst, Resulting in Gastric Fundus Varices: An Unusual Cause of Variceal Bleeding

**DOI:** 10.1155/1993/94170

**Published:** 1993

**Authors:** I. J. Klompmaker, E. B. Haagsma, M. J. H. Slooff

**Affiliations:** 1 Department of Internal Medicine University Hospital Oostersingel 59 Groningen 9713 EZ The Netherlands; 2 Department of Surgery University Hospital Groningen The Netherlands

## Abstract

A 27-year old female patient, born and living in Morocco, suffered from severe variceal bleeding and weight loss. Eventually a diagnosis of gastrict fundus varices was made supposedly resulting from partial obstruction of the splenic vein and collateral blood flow via the short gastric veins. Splenectomy was proposed. Peroperatively it became apparent that a solitary ecchinococcal cyst in the hilus of the spleen obstructed the blood flow in the splenic vein. The cyst, the spleen, and part of the pancreatic tail were removed. After splenectomy bleeding did not recur.


*HPB Surgery*, 1993, Vol. 6, pp. 229–233
Reprints available directly from the publisher
Photocopying permitted by license only

© 1993 Harwood Academic Publishers GmbH
Printed in the United States of America

### CASE REPORT

### SPLENIC VEIN OBSTRUCTION DUE TO A SOLITARY ECHINOCOCCAL SPLENIC CYST, RESULTING IN GASTRIC FUNDUS VARICES: AN UNUSUAL CAUSE OF VARICEAL BLEEDING

I.J. KLOMPMAKER,[1] E.B. HAAGSMA[1] and M.J.H. SLOOFF[2]

*Department of Internal Medicine[1] and Department of Surgery[2], University Hospital, Groningen, The Netherlands*

*(Received 31 October 1991)*

A 27-year old female patient, born and living in Morocco, suffered from severe variceal bleeding and weight loss. Eventually a diagnosis of gastrict fundus varices was made supposedly resulting from partial obstruction of the splenic vein and collateral blood flow via the short gastric veins. Splenectomy was proposed. Peroperatively it became apparent that a solitary ecchinococcal cyst in the hilus of the spleen obstructed the blood flow in the splenic vein. The cyst, the spleen, and párt of the pancreatic tail were removed. After splenectomy bleeding did not recur.

KEY WORDS: Solitary echinococcal splenic cyst, splenic vein obstruction, segmental portal hypertension

### INTRODUCTION

Isolated splenic vein occlusion or obstruction is uncommon[1–6]. Most frequently it is the result of inflammation or neoplasms of the pancreas. Sutton *et al.*[2] reported that in the majority of cases the causative factors are unknown. A more recent overview[6] counts only six idiopathic cases, but in 22 out of 209 cases the exact cause was not stated. In this large review only one patient had a hydatid cyst in the spleen. We also report a patient with splenic vein obstruction due to a solitary echinococcal cyst in the hilus of the spleen. This cyst resulted in gastric varices via the short gastric veins and variceal bleeding.

Echinococcal disease is endemic in certain countries. In Europe it is mostly found in the Mediterranean area[7]. Echinococcal cysts are often found in the liver. Extra-hepatic manifestations of the disease are not uncommon, but far less frequent[8]. Even in endemic countries making a diagnosis can be difficult, especially when the presenting symptoms are atypical. A non typical disease pattern in a non endemic area may even raise a more severe diagnostic and therapeutic pitfall.

---

Address correspondence to: I.J. Klompmaker MD, Dept. Internal Medicine, University Hospital, Oostersingel 59, 9713 EZ Groningen, The Netherlands

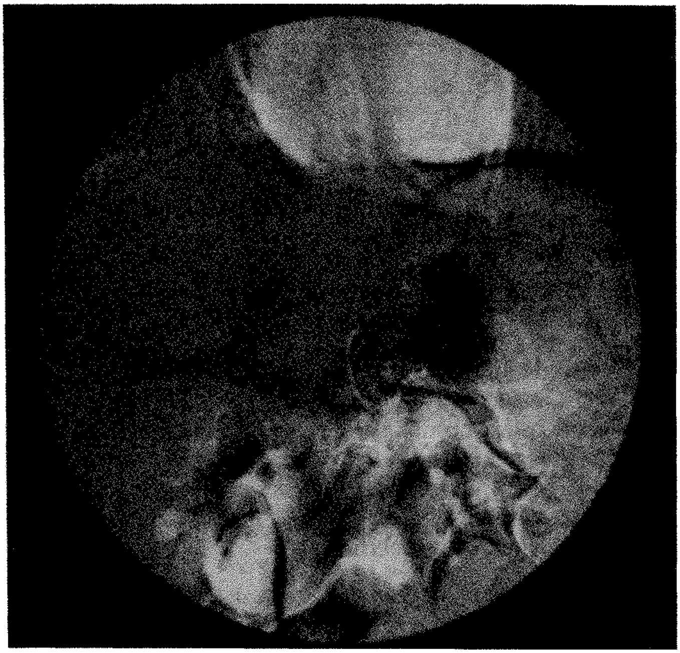

**Figure 1** Venous phase of selective splenic artery injection. Collateral flow via short gastric veins and narrowing of the splenic vein or collateral blood flow.

liver tests and synthesis function and/or normal liver biopsy should suggest portal vein or splenic vein thrombosis. Most cases of splenic vein thrombosis occur in association with pancreatic disease[5,6]. Isolated splenic vein thrombosis is uncommon[1–6]. Grunert[1] reported only 4 cases with isolated splenic vein occlusion (1.2%) in a series of 327 cases of portal thrombosis. Sutton *et al.* collected 54 documented isolated splenic vein thromboses in the English literature from 1900 till 1968[5]. Madsen *et al.* screened the literature till 1985 and found a total of 209 cases[6]. In both studies splenomegaly was reported in over 60% of the patients (Table 1). A high percentage of the reported cases had complications of upper gastrointestinal bleeding. Splenic vein occlusion is best established in the venous phase of upper abdominal arteriography[2,3,9]. Nowadays duplex ultrasound may be a useful diagnostic tool, but in case of collateral vessels in the hilum of liver or spleen it may not be 100% reliable.

Splenic vein obstruction in our patient appeared to be caused by a solitary echinococcal cyst in the hilum of the spleen. This resulted in "localised portal hypertension". Collateral flow via the short gastric veins developed gastric varices, which eventually bled repeatedly. This diagnosis was made only after opening the abdomen. Only in 5% of a large series of 179 patients with echinococcal disease was the spleen involved as the single organ[8]. Portal hypertension resulting in variceal bleeding due to echinococcal disease is mostly reported as a result of involvement

**Table 1** Etiology and main symptoms in cases of isolated splenic vein thrombosis according to Sutton[5] and Madsen[6]

| | *Sutton* | *Madsen* |
|---|---|---|
| Pancreatitis/neoplasm | 56% | 83% |
| Cause unknown or not stated | 30% | 13% |
| Splenomegaly | 65% | 71% |
| Variceal bleeding | 65% | 72% |

of the portal vein[8,10,11]. The association between hydatid disease and isolated splenic vein obstruction was only once reported[6].

In case of isolated splenic vein thrombosis, the therapy of choice is splenectomy[2–6,9]. This effectively stops the collateral blood flow to the gastric varices. Sutton reports splenectomy as therapy in 22 out of 54 collected cases[5]. Two patients rebled, but these cases were complicated by concomittant disease. In our patient the existence of an echinococcal cyst in the hilum of the spleen was an additional reason for removing the spleen. Three years after splenectomy, our patient is well without signs of rebleeding.

*Acknowledgement*
We are indebted to Miss E.J. Klompmaker for assistance in editing the manuscript.

### *References*

1. Grunert, R.D., Oeff, K., Gerstenberg, E. *et al.* (1966) Diagnosis of isolated splenic vein occlusion by radioportography. *Surgery*, **59**, 364–367
2. Yale, C.E. and Crummy, A.B. (1971) Splenic vein thrombosis and bleeding esophageal varices. *JAMA*, **217**, 317–320
3. Salam, A.A., Warren, W.D. and Tyras, D.H. (1973) Splenic vein thrombosis: a diagnosable and curable form of portal hypertension. *Surgery*, **74**, 961–972
4. Simpson, W.G., Schwartz, R.W. and Strodel, W.E. (1990) Splenic vein thrombosis. *South Med. J.*, **83**, 417–421
5. Sutton, J.P., Yarborough, D.Y. and Richards, J.T. (1970) Isolated splenic vein occlusion. *Arch. Surg.*, **100**, 623–626
6. Madsen, M.S., Petersen, T.H. and Sommer, H. (1986) Segmental portal hypertension. *Ann. Surg.*, **204**, 72–74
7. Lewis, J.W., Koss, N. and Kerstein, M.D. (1975) A review of echinococcal disease. *Ann. Surg.*, **181**, 390–396
8. Amir-Jahed, K.D., Fardin, R., Farzad, A.D. and Bakshandeh, K. (1975) Clinical echinococcosus. *Ann. Surg.*, **182**, 541–546
9. Boyer, T.D. (19??) Portal hypertension and its complications. In: *Hepatology. A textbook of liver disease*, pp. 477–478. Zakim and Boyer Eds. W.B. Saunders Co.; Philadelphia
10. Klein, Chr., Reikowski, H., Mueting, D., Matzander, U., Seebach, B. von (1971) Kasuistischer Beitrag zur Klinik des Leberechinokokkus. *Med. Welt.*, **22**, 1647–1650
11. Miguet, J.P., Bresson-Hadni, S., Becker, M.C., Lenys, D., Didier, D., Rohmer, P., Weill, F., Vuitton, D. and Gillet, M. (1988) L'echinococcose alveolaire hepatique. Donn<ea>s cliniques et morphologiques a propos de 80 cas. *Rev. Med. Suisse Romande*, **109**, 95–96

(*Accepted by S. Bengmark 18 December 1991*)

### INVITED COMMENTARY

This short paper reports a most unusual presentation of a hydatid cyst. Most surgeons dealing with hydatid disease will have seen splenic hydatids, and some will have seen cysts in the pancreas. The complication of splenic vein thrombosis must be rare indeed.

It is puzzling in this case that the ultrasound did not suggest a diagnosis of pancreatic cyst or even a more specific diagnosis of hydatid cysts. The nature of the "tubular structure" in the region of the splenic vein is not clear from the text, and one wonders whether this was part of the cyst. It would be helpful to know whether the hydatid was viable or whether it was an effete or dead hydatid. Severe pericystic inflammation tends to occur as the cyst dies, and the cyst contents change in consistency from watery clear to a milky consistency and finally to the consistency of toothpaste. Ultrasound is particularly good at detecting cysts with clear fluid, but mistakes may be made when the contents are thicker.

Whatever the reason for the failure of ultrasound to make the diagnosis preoperatively, it is clear from this paper that hydatids can cause unusual illnesses in people from hydatid endemic areas. Once again, it confirms the need for all hepatobiliary and pancreatic surgeons to be able to recognise and deal with the disease, whether they practise in endemic regions or not. The world has become a smaller place, and the hydatid cyst will travel comfortably with its host to any part of the world.

J.M. Little

